# Machine Learning Integrating Quantitative MRI and Clinical Features Predicts Loss of Ambulation in Duchenne Muscular Dystrophy

**DOI:** 10.1002/jcsm.70413

**Published:** 2026-10-22

**Authors:** Chuan Liu, Yu Song, Jiayu Wan, Ting Xu, Xinyuan Zhang, Yiqiao Yan, Hang Fu, Xiaotang Cai, Yingkun Guo, Yun Wang, Huayan Xu

**Affiliations:** ^1^ Department of Radiology, Medical Imaging Center, China Key Laboratory of Birth Defects and Related Diseases of Women and Children, Ministry of Education West China Second University Hospital, Sichuan University Chengdu China; ^2^ Department of Rehabilitation and Department of Pediatric Neurology, China Key Laboratory of Birth Defects and Related Diseases of Women and Children, Ministry of Education West China Second University Hospital, Sichuan University Chengdu China

**Keywords:** Duchenne muscular dystrophy, fat fraction, loss of ambulation, machine learning, quantitative MRI, random survival forest

## Abstract

**Background:**

Loss of ambulation (LOA) is a key milestone in Duchenne muscular dystrophy (DMD) with highly variable timing, complicating clinical counselling and trial design. Quantitative MRI non‐invasively assesses muscle fat infiltration, yet few models have integrated MRI with clinical variables for individualized LOA prediction. We aimed to develop and validate a machine‐learning model integrating quantitative MRI and clinical features to predict time to LOA and to derive interpretable, individualized risk estimates and candidate thresholds.

**Methods:**

This prospective study enrolled 188 boys with DMD (median age 9.10 years). Quantitative MRI parameters (T1, T2, fat fraction [FF]) of 19 hip/thigh muscles and clinical variables were measured. Patients were randomly split into training (80%) and test (20%) sets. Feature selection used LASSO, Boruta and recursive feature elimination based on random survival forest (RSF). Three survival models were compared: RSF and a ridge‐penalised Cox model (both integrating clinical and MRI‐derived features) and a multivariable Cox model restricted to clinical variables. Performance was assessed by concordance index (C‐index), time‐dependent area under the curve (AUC), integrated Brier score (IBS) and decision curve analysis at 12, 24, 36 and 48 months.

**Results:**

Over median 43.5 months follow‐up, 66 patients (35.1%) experienced LOA. Six predictors were retained: NSAA score, MRC%, age and three FF parameters (rectus femoris, vastus lateralis, obturator internus). In the test set (*n* = 40, 15 events), RSF achieved a C‐index of 0.870 (95% CI, 0.764–0.957), outperforming the clinical Cox (0.804) and ridge Cox (0.853) model. Time‐dependent AUCs of the RSF model were 0.984, 0.971, 0.913 and 0.850 at 12, 24, 36 and 48 months. RSF showed the best calibration, with the lowest IBS (0.076, 95% CI: 0.052–0.111), followed closely by the ridge Cox model (0.078) and the clinical Cox model (0.109). RSF stratified patients into low‐risk, intermediate‐risk and high‐risk groups; no LOA events occurred in the low‐risk group, with annual event rates were 7.2% and 34.6% in the intermediate‐ and high‐risk groups (log‐rank *p* = 0.0025). SHAP analysis identified exploratory thresholds (NSAA < 14.55, MRC% < 68.36%, rectus femoris FF > 32.30%, vastus lateralis FF > 58.00%, obturator internus FF > 7.43%). An interactive web application enables individualized risk prediction (https://westcndmd‐timer‐loa‐risk‐prediction‐tool‐8cygtrrbykvd9snmfcnhrp.streamlit.app/).

**Conclusions:**

A machine‐learning‐based RSF model integrating quantitative MRI FF metrics and clinical variables exhibited favourable predictive performance for time to LOA in DMD across short‐ and long‐term horizons. It provided interpretable, individualized risk estimates and was deployed as an online tool to support clinical decision‐making and trial enrichment.

## 1 Introduction

Duchenne muscular dystrophy (DMD) is an X‐linked recessive neuromuscular disorder resulting from insufficient or absent dystrophin production secondary to mutations in the X‐chromosomal DMD gene [1, 2]. In the absence of functional dystrophin, skeletal muscles undergo chronic damage, followed by progressive fibrosis and fatty infiltration, ultimately leading to motor weakness and irreversible loss of functional capacity. Loss of ambulation (LOA) represents a pivotal clinical milestone associated with accelerated disease progression and poorer patient outcomes and has a substantial impact on the physical, social and emotional well‐being of patients and their families [3, 4].

Despite advances in symptomatic management, the multifactorial nature of LOA progression in DMD [5] leads to substantial interindividual variability in its timing and trajectory, posing major challenges for clinical counselling, therapeutic decision‐making and trial design [6]. Functional parameters measured by clinical assessments, such as the North Star Ambulatory Assessment (NSAA), 6‐min walk test and timed motor function tests, have been widely used in DMD studies [7, 8]. However, these tests are effort‐dependent and require active patient cooperation, which can be particularly challenging in children. Notably, NSAA trajectories show wide inter‐subject and intra‐subject variability due to differences in glucocorticoid regimens, genotype variation and variability in test administration and patient performance [9]. These limitations highlight the need for objective, quantitative biomarkers that can complement traditional clinical assessments. Quantitative muscle MRI has emerged as a promising biomarker, enabling precise quantification of fat infiltration, edema and muscle volume loss [10, 11, 12]. It can predict future functional changes and serve as surrogate endpoints for clinical trials [13, 14, 15]. Nevertheless, most studies have focused on diagnostic value or single‐factor associations and few have integrated high‐dimensional MRI features with clinical variables for longitudinal outcome prediction.

Early LOA prediction could inform timely clinical decisions, such as optimizing glucocorticoid regimens and implementing multidisciplinary rehabilitation, potentially prolonging ambulation and improving quality of life. Machine learning (ML) has been increasingly applied to medical prediction tasks and may improve predictive performance compared with conventional statistical methods [16]. ML approaches have also been successfully used in other rare diseases [17, 18, 19]. Among these methods, the random survival forest (RSF) model dispenses with assumptions regarding survival time distribution and proportional hazards, enables effective processing of censored data and may reduce overfitting and convergence issues inherent to conventional Cox regression approaches. RSF has demonstrated great advantages in survival analysis and prediction. Yet, the feasibility of ML, especially RSF for LOA prediction in DMD remains underexplored.

Therefore, our primary aim was to develop and validate an RSF‐based ML predictive model by integrating multiparametric quantitative MRI with clinical features to forecast time to LOA and to provide interpretable, individualized risk estimates with candidate risk thresholds to guide clinical decision‐making. Recognizing that predictive accuracy may vary across the disease trajectory, we evaluated the RSF model's performance across short‐term (12‐month), medium‐term (24‐month) and long‐term (36‐ and 48‐month) horizons, providing a tailored analytical framework for clinical decision‐making. For secondary benchmarking purposes, we compared performance against conventional Cox‐based survival models to disentangle the incremental prognostic value of MRI biomarkers from gains conferred by the machine‐learning algorithm.

## 2 Methods

### 2.1 Study Population

Patient recruitment was conducted as part of a prospective longitudinal single‐centre cohort study investigating DMD muscular injury and cardiomyopathy (clinical trial registration: https://www.chictr.org.cn/searchproj.html https://www.chictr.org.cn/showproj.html?proj=141024; registration Nos. ChiCTR1800018340 and ChiCTR2200055651). Between March 2020 and February 2023, 447 boys with genetically and/or pathologically confirmed DMD were enrolled. Exclusion criteria included the following: (a) substantial missing data in baseline medical records; (b) absence of quantitative MRI examination; (c) any disease‐modifying DMD therapies (e.g., gene therapy, exon skipping) at baseline; and (d) presence of LOA at the initial visit. LOA was defined as the inability to complete an unassisted 10‐m walk/run within 45 s or the need for long‐term wheelchair use for more than 3 months [4, 5]. The study was approved by the institutional ethics committee and written informed consent was obtained from all guardians.

### 2.2 Muscle Quantitative MRI Protocol

Imaging was performed using a 3‐tesla (T) MR scanner (Magnetom Skyra, Siemens Healthineers, Erlangen, Germany) with an 18‐channel receiver coil. All patients underwent MRI from the iliac crest to the mid‐thigh, with scanning protocols including T1 mapping, T2 mapping and Dixon sequences. Sequence parameters were set as follows: T1 mapping was performed using a modified Look–Locker inversion recovery (MOLLI) sequence, with echo time (TE) = 1.1 ms, repetition time (TR) = 2.7 ms, flip angle (FA) = 35°, slice thickness = 6 mm, matrix = 400 × 340 and acquisition time (T_acq_) = 54 s; T2 mapping was performed using a three‐echo sequence (TE = 0/30/55 ms), with TR = 3 ms, FA = 12°, slice thickness = 6 mm, matrix = 410 × 330 and T_acq_ = 54 s; quantitative water/fat imaging (T2‐weighted Dixon sequence) was performed using a fast spin‐echo sequence, with TE = 55 ms, TR = 3660 ms, FA = 150°, slice thickness = 6 mm, matrix = 360 × 360 and T_acq_ = 2 min 34 s. The interval between performance of MRI and the corresponding outpatient visit was restricted to no more than 1 month.

### 2.3 Clinical Indicator Acquisition and MRI Image Analysis

Demographic characteristics, laboratory measurements and clinical variables were retrieved from electronic medical records and standardized questionnaires, including age, height, weight, mutation type, glucocorticoid use at baseline, fracture status, bone mineral density value, Medical Research Council muscle strength percentage (MRC%), NSAA, creatine kinase and creatine kinase myocardial band. All quantitative MRI images were analysed using Siemens MR Post‐Processing workstation (Syngo Via, Erlangen, Germany) by 2 radiologists (Y.S. a radiologist with 5 years of experience and T.X., a radiologist with 3 years of experience), blinded to clinical data. In cases of discordance, consensus was reached through discussion between the two observers.

Quantitative MRI parameters, including T1 relaxation time, T2 relaxation time and fat fraction (FF), were measured for 19 individual muscles of the hip and thigh. These muscles comprised: gluteus maximus, gluteus medius, gluteus minimus, iliacus, tensor fasciae latae, obturator internus, sartorius, pectineus, semitendinosus, semimembranosus, gracilis, adductor magnus, adductor longus, adductor brevis, rectus femoris, vastus lateralis, vastus intermedius, vastus medialis and biceps femoris long head. Slices that contained the largest area of visible muscle with good differentiation of diverse muscle groups were chosen for placing the regions of interest (ROI), including four major cross‐sectional levels [11]: (1) level near the sciatic foramen; (2) level near the greater trochanter‐ischial tuberosity; (3) level near the proximal part of the femoral diaphysis; and (4) level near the middle of the femoral diaphysis (Figure S1). T1 and T2 maps were subjected to pixel‐wise colour coding, with hues corresponding to respective T1 and T2 value ranges. Placing each muscle's ROI on the corresponding map enabled automated calculation of mean T1 or T2 values. Fat fraction was computed as [signal intensity (SI) fat / (SI fat + SI water)] × 100% using reconstructed fat‐water images [15]. The size of the ROI was determined by using the individual muscle size on the axial images. The average T1, T2 and fat fraction values were calculated using the ROIs outlined on three consecutive slices of each muscle.

### 2.4 Follow‐Up

For each participant, the first visit with complete data for key variables was designated as the baseline assessment. Outcome events were ascertained through semi‐annual follow‐up assessments conducted until October 2025 via clinic visits, telephone interviews and medical record review. Time to LOA (months) was calculated from the baseline visit to the date of LOA occurrence or the last follow‐up.

### 2.5 Data Preprocessing

The dataset was randomly split into training (80%) and test (20%) cohorts prior to any imputation. Variables with ≥ 20% missing values were excluded a priori based on the training‐set data. Remaining missing values were imputed using the missForest random‐forest algorithm [20], which was fitted exclusively on the training set. The trained imputation model was subsequently applied to the test set to avoid data leakage. Feature selection was performed exclusively on the training set. For feature standardization, means and standard deviations were calculated from the training set and subsequently applied to normalize both training and test sets.

### 2.6 Feature Selection

Candidate clinical features were preselected using univariate Cox regression with a threshold of *p* < 0.05. All variables meeting this criterion were retained as potential predictors, as the subsequent random survival forest algorithm is relatively robust to correlated inputs. For quantitative MRI features (fat fraction, T1, T2 of 19 hip and thigh muscles), a three‐step dimensionality reduction was conducted exclusively on the training set: (1) LASSO regression (*α* = 1) with 5‐fold cross‐validation (CV) to select features with nonzero coefficients at λ.min; (2) Boruta algorithm, a random forest‐based wrapper, to identify features whose importance significantly exceeded that of shadow features; (3) Recursive feature elimination (RFE) using RSF as the estimator and five‐fold CV to select the optimal number of features. The final quantitative MRI feature set was defined as the intersection of features selected by all three methods.

### 2.7 Model Construction

Three survival prediction models were built using the selected features. (i) The clinical Cox model was a multivariable Cox proportional hazards regression including three clinical variables retained after univariate screening: NSAA score, MRC% and age. (ii) The RSF model integrated the three clinical variables together with the final quantitative MRI features selected by the three‐step procedure. Hyperparameters were tuned by grid search with five‐fold CV on the training set. The optimal hyperparameter combination, which yielded the highest cross‐validated C‐index, was max_features = 3 and min_samples_split = 15 (mean CV C‐index = 0.890). The final model was refitted with 1000 trees, the log‐rank splitting rule and a fixed random state of 123. (iii) The ridge‐penalised Cox model utilized the same six predictors as the RSF, with all features standardized using training‐set statistics. The optimal L2 penalty (λ = 0.02) was determined via 5‐fold CV (mean CV C‐index = 0.897) and the final model was fitted on the full training dataset. The ridge model was implemented to mitigate collinearity among the correlated FF features. Discrimination was assessed using the C‐index and time‐dependent area under the curve (AUC) at 12 months (short‐term), 24 months (medium‐term) and 36 and 48 months (long‐term), with 95% confidence intervals derived from 200 bootstrap resamples. Model calibration was assessed using the inverse‐probability‐of‐censoring‐weighted integrated Brier score (IBS). Clinical utility was evaluated by decision curve analysis (DCA) at each predefined time point across threshold probabilities ranging from 0 to 0.5.

### 2.8 Model Interpretation and Deployment

SHAP analysis [21] was performed on the individual‐level hazard risk scores derived from the trained RSF model, generating feature importance rankings, dependence plots with LOWESS smoothing and individual waterfall plots. To explore time‐varying feature importance, we performed SurvSHAP(t) analysis using the survex package. Mean absolute SHAP values were computed at all unique event times, generating temporal importance curves for each predictor. For risk stratification, RSF‐derived individual hazard risk scores from the training set were divided into tertiles (low, intermediate, high risk); these pre‐defined tertile cutoffs were applied to the test set and Kaplan–Meier curves with log‐rank tests compared LOA‐free survival among the three groups. Additionally, for each risk group we calculated the sample size, the annual event rate (per 100 person‐years) and the rate difference (RD) relative to the low‐risk group. These results were presented in a combined bar‐line graph. The final RSF model was exported and deployed as an interactive web application using Streamlit, named DMD‐TIMER (time‐to‐LOA integrated multiparameter estimation with random survival forest), which integrates multiparametric quantitative MRI with clinical features to forecast time to LOA.

### 2.9 Statistical Analysis

Statistical analyses were performed using Python 3.9 and R 4.4.2. For continuous data, the Shapiro–Wilk test for normality was first performed. For data that followed a normal distribution with equal variances, independent *t*‐tests were conducted and results were presented as mean ± standard deviation. Non‐normally distributed continuous variables were compared using the Mann–Whitney U test and are presented as the median and interquartile range. Categorical data were presented as frequencies and percentages, with group differences assessed using the chi‐square test or Fisher's exact test. Statistical significance was defined as a two‐sided *p*‐value < 0.05.

## 3 Results

### 3.1 Patients Characteristics

A total of 447 patients with DMD were initially screened and 188 patients were eventually included (Figure 1). The median age of the cohort was 9.10 years (IQR, 7.90–10.20). The median follow‐up duration was 43.50 months (IQR, 23.00–54.00). During follow‐up, 66 patients (35.1%) experienced LOA. The cohort was randomly divided into a training set (*n* = 148, 51 events) and a test set (*n* = 40, 15 events). The two sets were well balanced in terms of demographic, clinical and quantitative MRI parameters (Table S1).

**FIGURE 1:**
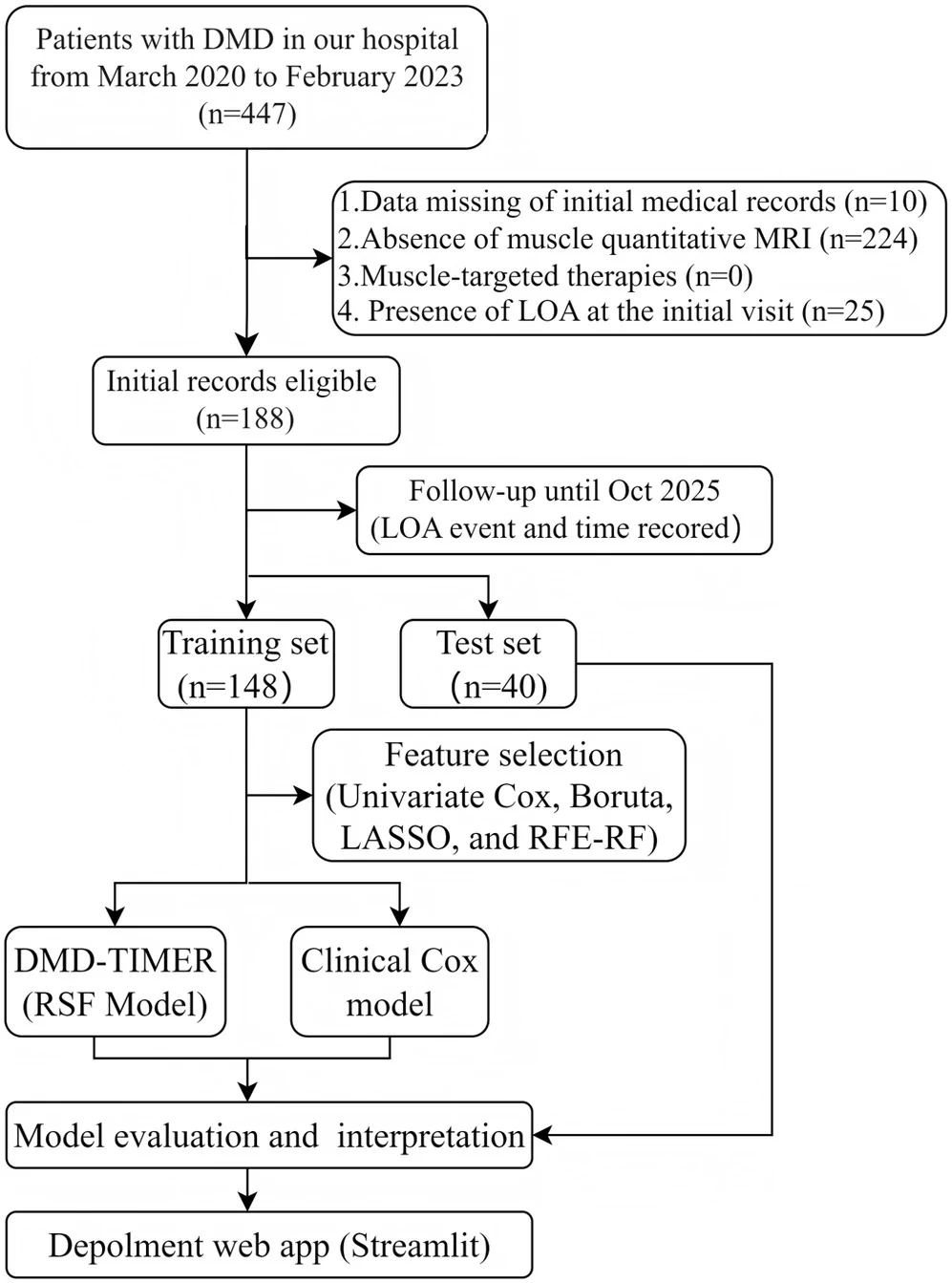
Flowchart of study design and patient selection.

### 3.2 Feature Selection

Univariate Cox regression identified three clinical variables significantly associated with LOA (*p* < 0.05): NSAA score, MRC% and age (Table S2). These three were retained as clinical predictors. In multivariate Cox analysis including all three, only NSAA score remained an independent predictor (HR = 0.845; 95% CI, 0.802–0.891; *p* < 0.001). For quantitative MRI features, three methods were used for screening. LASSO regression (*α* = 1) with five‐fold cross‐validation identified 11 key features (λ.min = 0.0586; Figure S2). The Boruta algorithm confirmed 12 features (Figure S3). RFE‐RSF selected three optimal features based on the highest C‐index (Figure S4). The intersection of LASSO, Boruta and RFE yielded three fat fraction (FF) parameters: rectus femoris FF, vastus lateralis FF and obturator internus FF (Figure S5). Notably, no T1 or T2 parameters survived the selection. These six variables (NSAA, MRC%, age and the three FF parameters) were used for subsequent ML model development.

### 3.3 Model Performance

Three survival models were developed and evaluated on both training and test sets (Table 1). In the training set, the RSF model demonstrated best calibration, with an IBS of 0.050 (95% CI, 0.037–0.061), closely followed by the ridge Cox model (0.070, 95% CI, 0.054–0.089) and the clinical Cox model (0.074, 95% CI, 0.058–0.092). The RSF model also showed the highest discrimination, achieving a C‐index of 0.932 (95% CI, 0.907–0.954), compared with 0.897 (95% CI, 0.860–0.929) for the ridge Cox model and 0.895 (95% CI, 0.858–0.926) for the clinical Cox model. Training‐set time‐dependent AUCs for the RSF model were 0.977 (95% CI: 0.950–0.998) at 12 months, 0.974 (95% CI: 0.949–0.992) at 24 months, 0.977 (95% CI, 0.951–0.996) at 36 months and 0.963 (95% CI, 0.930–0.989) at 48 months, outperforming both Cox models across all time points (Table 2; Figure 2; Figure S6). DCA at threshold probabilities ranging from 0 to 0.5 revealed that the RSF model yielded the highest net clinical benefit across the entire threshold range at all four horizons, with the advantage most pronounced at 48 months (Figure 3).

**TABLE 1:** Discrimination and prediction error of the three models in the training and test sets.

Dataset	Model	C‐index (95% CI)	IBS (95% CI)
Training set	RSF	0.932 (0.907–0.954)	0.050 (0.037–0.061)
	Clinical Cox	0.895 (0.858–0.926)	0.074 (0.058–0.092)
	Ridge Cox	0.897 (0.860–0.929)	0.070 (0.054–0.089)
Test set	RSF	0.870 (0.764–0.957)	0.076 (0.052–0.111)
	Clinical Cox	0.804 (0.685–0.899)	0.109 (0.061–0.170)
	Ridge Cox	0.853 (0.748–0.941)	0.078 (0.046–0.114)

*Note:* Values are point estimates with bootstrap 95% CIs (200 resamples, percentile method).

Abbreviations: CI, confidence interval; C‐index, concordance index; IBS, integrated Brier score; RSF, random survival forest.

**TABLE 2:** Time‐dependent area under the receiver operating characteristic curve of the three models at 12, 24, 36 and 48 months in the training and test sets.

Model	Training set				Test set			
	12 mo	24 mo	36 mo	48 mo	12 mo	24 mo	36 mo	48 mo
Clinical Cox	0.960 (0.920–0.996)	0.955 (0.919–0.984)	0.947 (0.904–0.981)	0.920 (0.876–0.964)	0.910 (0.811–1.000)	0.876 (0.755–0.982)	0.872 (0.752–0.975)	0.823 (0.653–0.955)
Ridge Cox	0.968 (0.934–0.993)	0.950 (0.908–0.980)	0.950 (0.910–0.979)	0.931 (0.878–0.969)	0.947 (0.848–1.000)	0.956 (0.886–1.000)	0.915 (0.806–0.996)	0.853 (0.697–0.979)
RSF	0.977 (0.950–0.998)	0.974 (0.949–0.992)	0.977 (0.951–0.996)	0.963 (0.930–0.989)	0.984 (0.929–1.000)	0.971 (0.911–1.000)	0.913 (0.805–0.997)	0.850 (0.701–0.973)

*Note:* Values are bootstrap mean AUCs with 95% CIs (200 resamples, percentile method).

Abbreviations: AUC, area under the curve; CI, confidence interval; mo, months.

**FIGURE 2:**
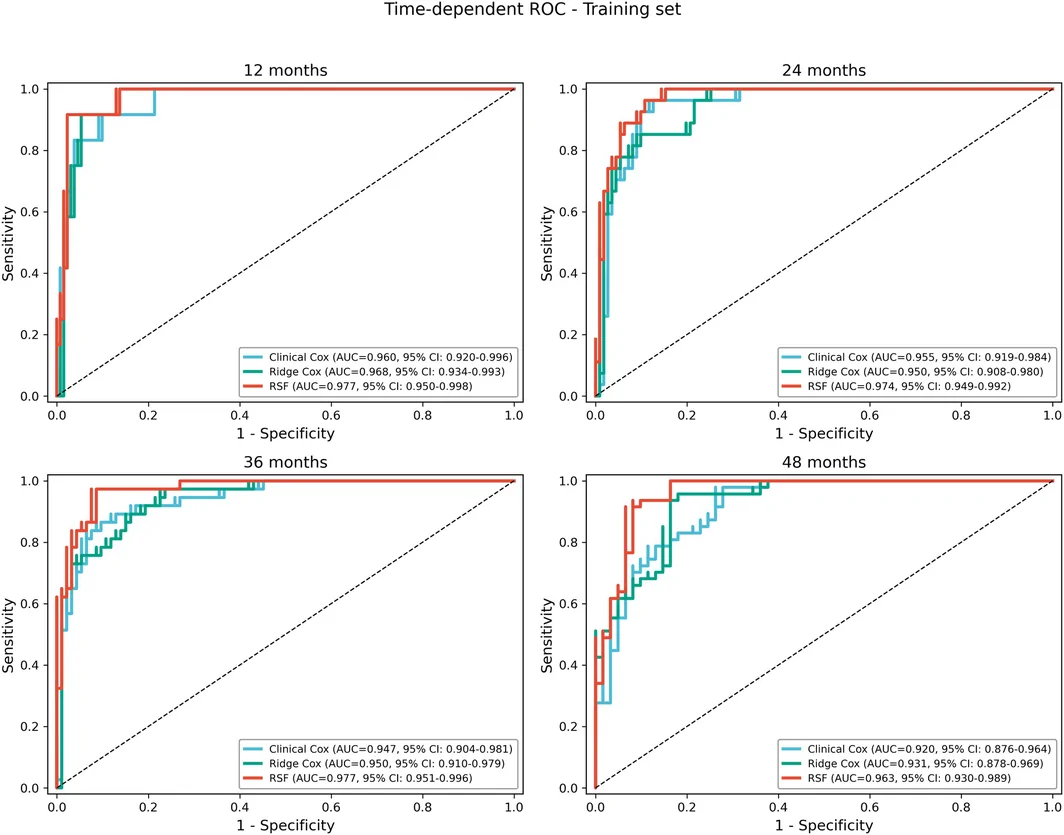
Time‐dependent area under the curve for the RSF, clinical Cox and ridge‐penalised Cox models in the training set.

**FIGURE 3:**
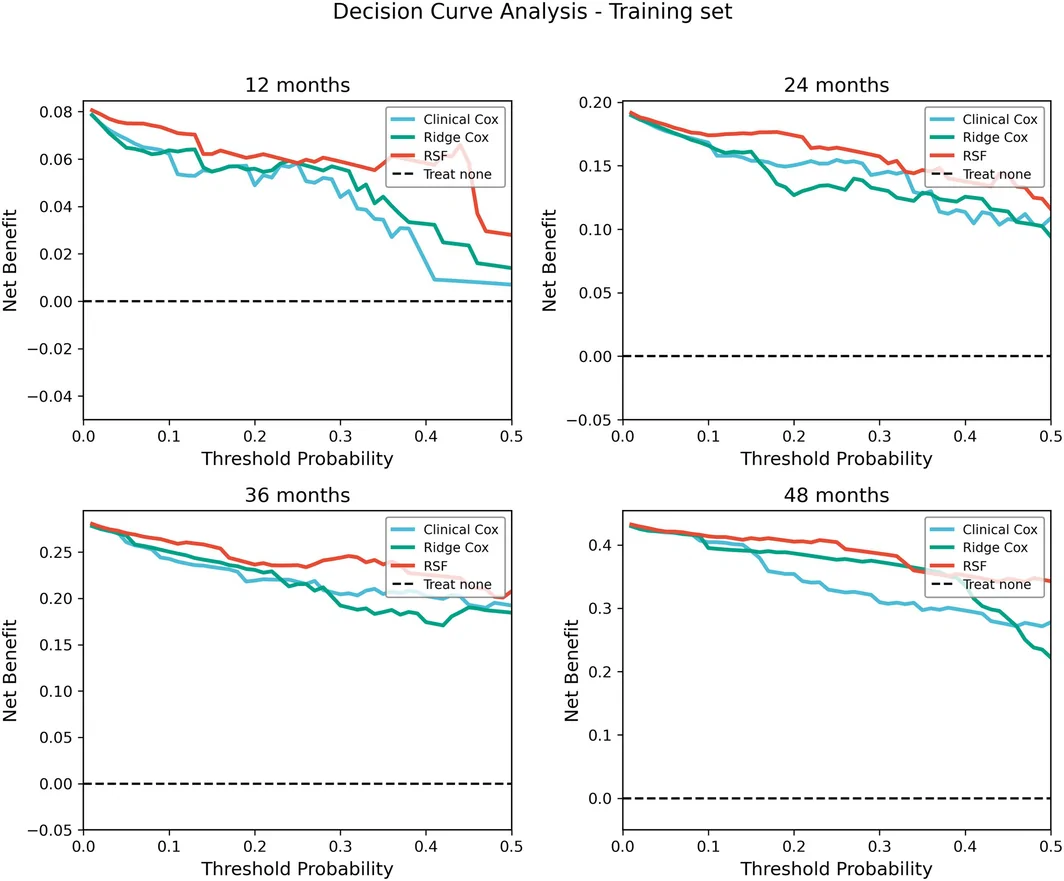
Decision curve analysis for the RSF, clinical Cox and ridge‐penalised Cox models in the training set.

In the test set, the RSF model maintained its superior performance, with a C‐index of 0.870 (95% CI, 0.764–0.957) versus 0.853 (95% CI, 0.748–0.941) for the ridge Cox model and 0.804 (95% CI, 0.685–0.899) for the Cox model (Table 1). With respect to the IBS, the RSF model achieved the best calibration (0.076, 95% CI, 0.052–0.111), with the ridge Cox model a close second (0.078, 95% CI, 0.046–0.114) and the clinical Cox model the highest (0.109, 95% CI, 0.061–0.170) (Table 1). Time‐dependent AUCs for the RSF model in test set were 0.984 (95% CI, 0.929–1.00) at 12 months, 0.971 (95% CI, 0.911–1.00) at 24 months, 0.913 (95% CI, 0.805–0.997) at 36 months and 0.850 (95% CI, 0.701–0.973) at 48 months, again outperforming the clinical Cox model at all horizons (Table 2; Figure S6; Figure S7). In the test set, the three models performed similarly at low threshold probabilities but diverged at higher thresholds (Figure S8). RSF yielded the highest, most stable net benefit within the clinically relevant threshold range.

### 3.4 Risk Stratification

Using continuous individual hazard scores derived from the trained RSF model, risk‐stratification cut‐offs were determined by tertiles within the training set, assigning training‐set patients to low‐risk (*n* = 50), intermediate‐risk (*n* = 49) and high‐risk (*n* = 49) subgroups. To prevent data leakage, these training‐set‐derived cut‐offs were directly applied to the independent test set, generating low‐risk (*n* = 8), intermediate‐risk (*n* = 16) and high‐risk (*n* = 16) groups. Kaplan–Meier curves for the training set revealed a clear separation among the three risk groups (log‐rank *p* < 0.0001; Figure 4A). In the test set, the log‐rank test also showed a significant difference (*p* = 0.0025; Figure 4C). To further quantify absolute risk, we calculated the annual event rate per 100 person‐years for each risk group within the observed follow‐up period. In the training set, the observed numbers of LOA events in the low‐risk, intermediate‐risk and high‐risk groups were 0, 8 and 43, respectively. No LOA events were observed in the low‐risk group (exact Poisson 95% upper bound ≈ 1.7 per 100 person‐years), with annual event rates of 8.2% and 42.4% for the intermediate‐ and high‐risk groups. The incidence‐rate differences (RD) versus the low‐risk group were 8.2% for the intermediate‐risk group and 42.4% for the high‐risk group (Figure 4B). In the test set, events were 0, 3 and 12. No LOA events were observed in the low‐risk groups (exact Poisson 95% upper bound ≈ 10.3 per 100 person‐years); annual event rates were 7.2% and 34.6%, with corresponding RDs of 7.2% and 34.6% (Figure 4D). A consistent risk gradient was observed across both cohorts, confirming that the RSF model effectively discriminates patients at distinct risk of LOA and provides clinically meaningful absolute risk estimates.

**FIGURE 4:**
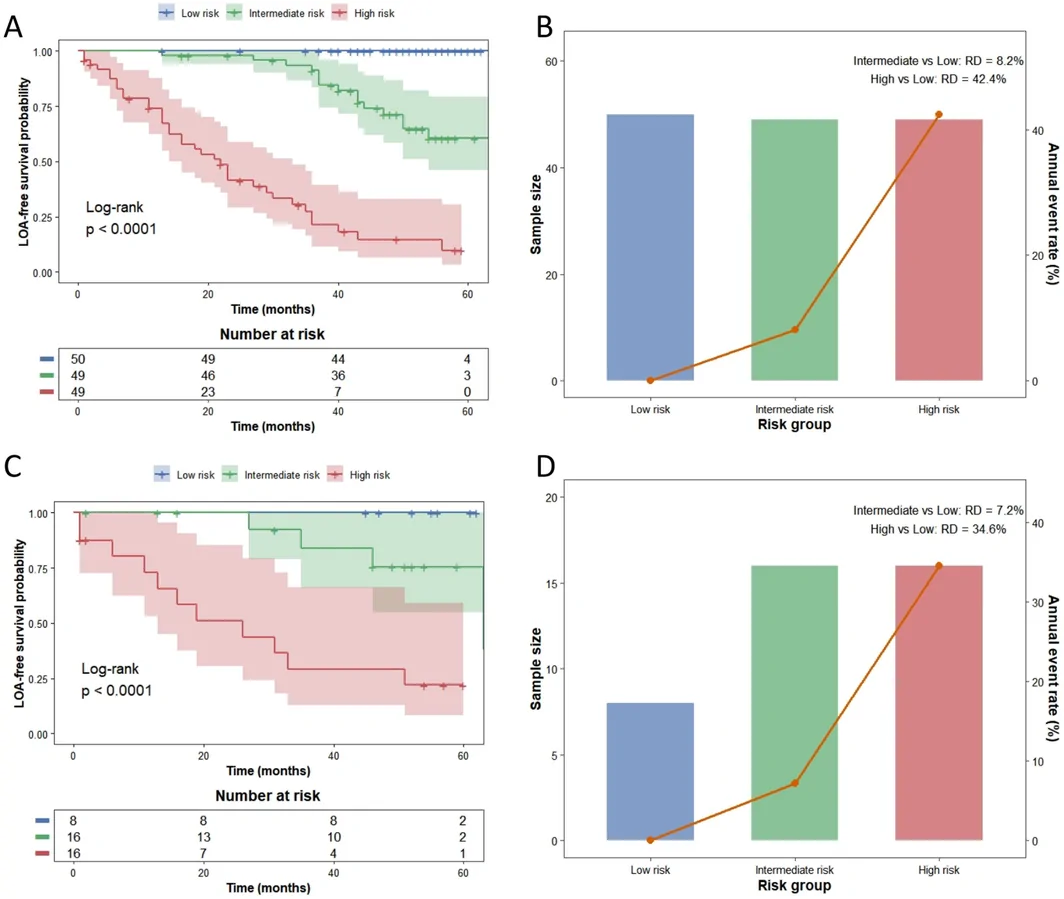
RSF‐based risk stratification in the training (A, B) and test (C, D) sets. (A, C) Kaplan–Meier curves with numbers at risk at baseline and follow‐up intervals (training: *p* < 0.0001; test: *p* = 0.0025, log‐rank). (B, D) Annual event rates (per 100 person‐years) and rate differences (RD) vs. low‐risk group. Total LOA events: training—0 (low), 8 (intermediate), 43 (high); test—0, 3, 12. Exact Poisson 95% upper bounds for annual event rate in the zero‐event low‐risk group: training ≈1.7, test ≈10.3 per 100 person‐years (approximated from median follow‐up). As zero events in low‐risk group precluded HR/RR estimation, RD and log‐rank P values are presented as primary effect measures.

### 3.5 Model Interpretation

SHAP feature importance analysis (Figure S9) ranked NSAA score as the most influential predictor of LOA, followed by the three imaging fat fraction parameters (rectus femoris FF, vastus lateralis FF and obturator internus FF), MRC% and age. The beeswarm summary plot further illustrated that lower NSAA scores and higher fat fraction values in the assessed muscles were associated with increased LOA risk. SurvSHAP(t) analysis (Figure S8) showed that the importance of muscle fat fractions (rectus femoris, vastus lateralis, obturator internus) increased steadily over time, indicating their growing contribution to long‐term LOA risk. In contrast, NSAA Score importance peaked in the medium term and then declined, suggesting its strongest predictive value for mid‐range horizons but reduced utility as patients approach ambulation loss. MRC% remained stable with modest importance, whereas age consistently contributed negligibly.

SHAP dependence plots with LOWESS smoothing were generated to identify potential non‐linear relationships and exploratory, model‐derived candidate risk thresholds (Figure 5). For each continuous predictor, the point where the smoothed curve crosses zero (SHAP value = 0) was noted as a candidate threshold from our six‐feature RSF model. A threshold was identified for NSAA Score at 14.55, where scores lower than this value positively contributed to LOA risk (positive SHAP), whereas scores higher than 14.55 showed a protective negative SHAP effect. For muscle fat fractions, thresholds were identified at 32.30% for rectus femoris FF, 58.00% for vastus lateralis FF and 7.43% for obturator internus FF; exceeding these thresholds shifted SHAP values from negative to positive, indicating increased risk. MRC% showed a threshold at 68.36%, with lower percentages increasing risk. In contrast to the other predictors, age showed a nearly flat SHAP dependence curve, indicating a negligible role in LOA prediction; the zero‐crossings observed were not considered meaningful. SHAP waterfall plots for representative patients from each risk group further illustrated how individual feature values contribute to personalized LOA risk predictions (Figure S10).

**FIGURE 5:**
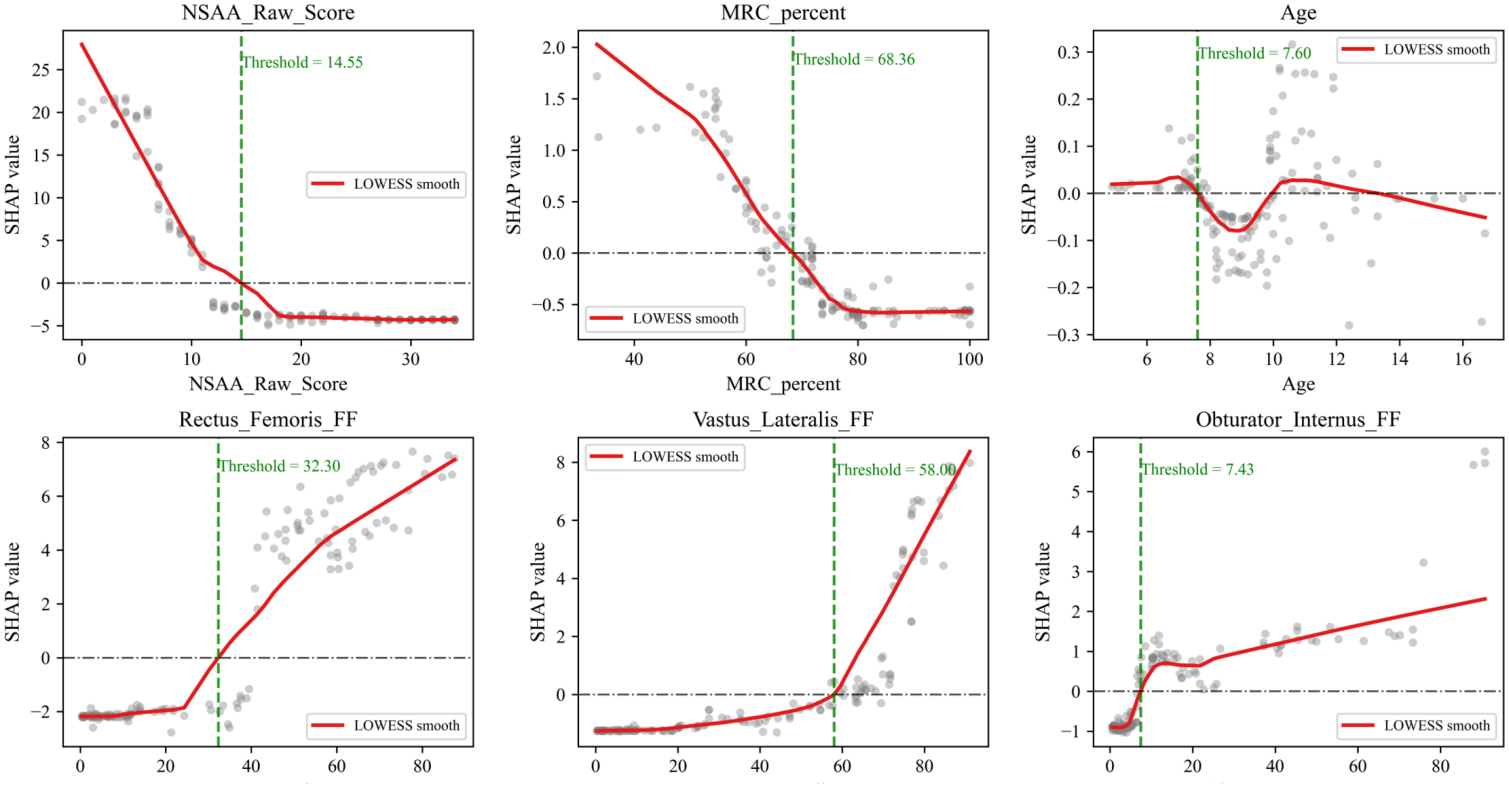
SHAP dependence plots with LOWESS smoothing identifying model‐derived exploratory candidate thresholds.

### 3.6 Online Prediction Tool

The final RSF model was deployed as an interactive web application using the Streamlit framework. Using this tool, clinicians and parents can input the six required parameters (NSAA score, MRC%, age and the three muscle fat fractions) into the DMD‐TIMER tool. The application then generates a personalized survival curve and calculates the predicted risk probability of LOA at 12, 24, 36 and 48 months (Figure 6). The tool is publicly accessible at https://westcndmd‐timer‐loa‐risk‐prediction‐tool‐8cygtrrbykvd9snmfcnhrp.streamlit.app/.

**FIGURE 6:**
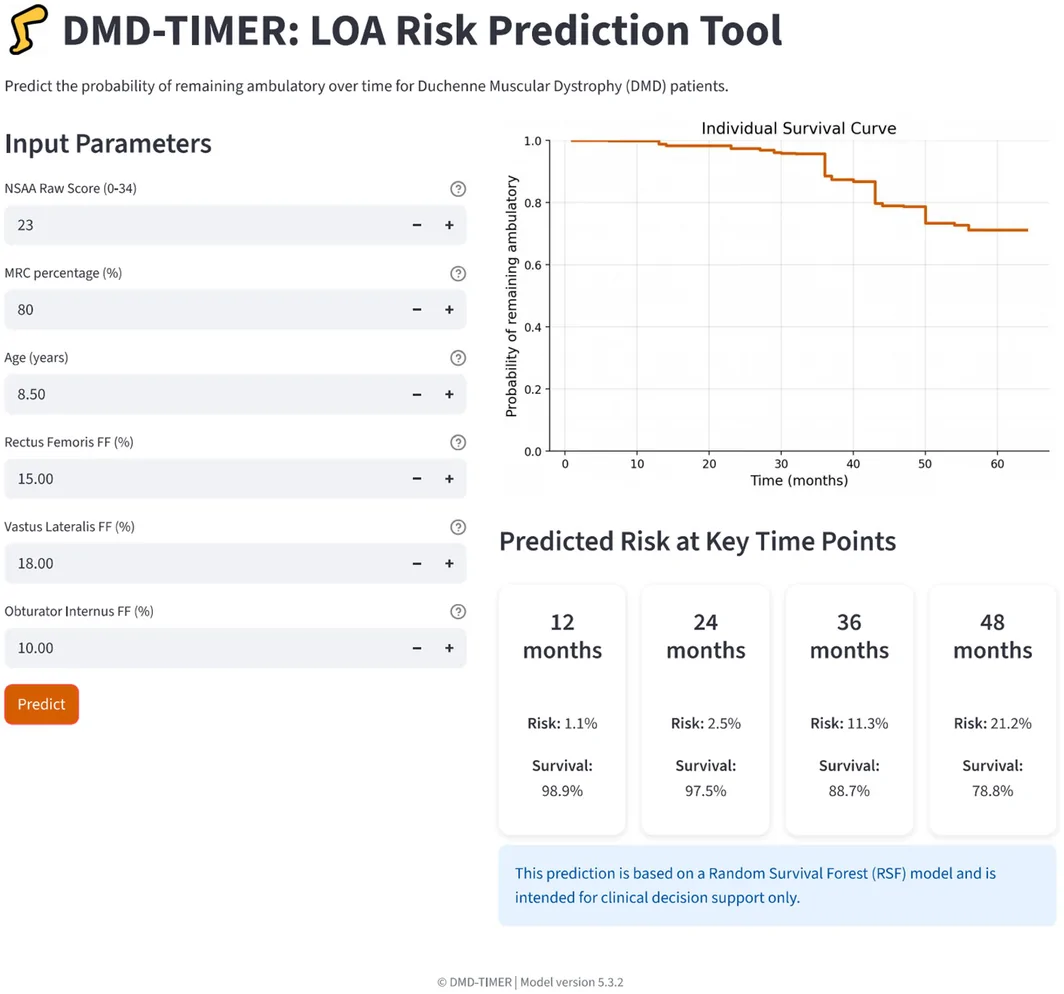
Interactive web application for individualized LOA risk prediction (DMD‐TIMER).

## 4 Discussion

In this study, we developed and validated a ML‐based RSF risk stratification tool, named DMD‐TIMER, which integrates three clinical variables (NSAA score, MRC%, age) and three quantitative MRI‐derived FF parameters (rectus femoris FF, vastus lateralis FF and obturator internus FF) to predict time to LOA in patients with DMD. Our study confirmed that MRI‐derived fat‐fraction measurements yield incremental prognostic information even within a conventional linear framework, whereas the further improvement seen with RSF suggests added discriminative power derived from modelling non‐linear effects and variable interactions. The RSF model demonstrated excellent predictive performance (test‐set C‐index, 0.870; time‐dependent AUCs 0.984 at 12 months, 0.971 at 24 months, 0.913 at 36 months and 0.850 at 48 months). Moreover, DMD‐TIMER effectively stratified patients into distinct low‐, intermediate‐ and high‐risk groups, with no LOA events observed in the low‐risk group and a consistent risk gradient in annual event rates (training: 8.2%, 42.4%; test: 7.2%, 34.6% in the intermediate‐ and high‐risk groups). These findings underscore the potential of DMD‐TIMER for personalized clinical decision‐making across short‐ and long‐term prediction horizons.

Among the clinical predictors, the dominant influence of the NSAA score on LOA prediction aligns with its well‐established prognostic value [8, 9]. The threshold we identified (NSAA score < 14.55) closely matches the mean score of 13/34 reported by Ricotti et al. 2 years before LOA [8], suggesting a clinically meaningful warning zone. For MRC%, the threshold of approximately 68.3% may provide a practical target for strength‐preserving interventions in clinical practice and trials. Importantly, although NSAA carried higher SHAP importance, MRC% offers complementary information that may be less subject to patient cooperation or ceiling effects. Together, these two clinical variables, when combined with quantitative MRI fat fraction, enable a more robust risk stratification than either clinical or imaging markers alone.

Among the MRI parameters, strikingly, only FF parameters survived the three‐step intersection of LASSO, Boruta and RFE‐RSF, whereas T1 and T2 relaxation times were not retained. This aligns with our previous findings that FF shows the highest responsiveness to disease progression and can detect subclinical progression even in functionally stable patients over 12‐month follow‐up [13, 22]. FF directly quantifies irreversible fatty infiltration, a core pathological hallmark of DMD [23, 24], whereas T1/T2 primarily reflect potentially reversible edema and inflammation, making them less predictive of a hard endpoint like LOA. Consistent with this, our work has shown that T1/T2 excel in early diagnosis, for example identifying DMD patients without detectable fat infiltration [11, 12, 25] and in differentiating DMD from Becker muscular dystrophy [26]. Among the three selected FF parameters, the SHAP importance ranking was consistent with the physiological expectation that muscles more involved in ambulation ranked higher. The inclusion of rectus femoris FF and vastus lateralis FF was expected, given their well‐documented role as key components of the quadriceps group in predicting functional decline in DMD [27, 28]. Beyond confirming their predictive role, our SHAP analysis based on our six‐feature RSF model further identified exploratory thresholds for these muscles: 32.30% for rectus femoris and 58.00% for vastus lateralis, above which LOA risk increased sharply. These thresholds may help clinicians identify patients at imminent risk. In contrast, the selection of obturator internus FF was unexpected. Although the role of this muscle in DMD has not been previously reported, anatomically it belongs to the hip deep external rotator group and functions synergistically with the abductors. Given our prior finding that abductor fat fraction independently predicts motor decline [13], the inclusion of obturator internus FF may reflect a systemic pattern of fatty infiltration extending beyond ambulatory muscles. The low threshold (7.43%) suggests it could be an early marker of disease activity, but this finding requires independent validation.

Although prior models have advanced prognostic assessment in DMD, several limitations remain. Goemans et al. [29, 30] developed multivariable regression models using generalized estimating equations to predict 1‐year changes in 6‐min walk distance and 4‐stair climb. They demonstrated that adding timed function tests (e.g., 10‐m walk/run, rise from supine, 4‐stair climb itself) to age, baseline functional measure and steroid use significantly improved prognostic accuracy for the 1‐year change. Hibma et al. [31] built a joint model linking NSAA to LOA, yet it remains fully parametric and lacks quantitative MRI biomarkers. Several studies have incorporated quantitative MRI to characterize disease progression [32, 33, 34], but none directly modelled time to LOA or provided individualized survival predictions. Our model directly addresses these gaps. One key strength of DMD‐TIMER is its ability to provide accurate predictions up to 48 months, supporting long‐term care planning such as timing of rehabilitation interventions, glucocorticoid dose adjustments and financial planning for rising care costs. Moreover, DMD‐TIMER effectively stratified patients into low‐, intermediate‐ and high‐risk groups, with a consistent risk gradient in annual event rates. Given the highly variable disease trajectory in DMD, where long‐term prognostic uncertainty imposes a heavy psychological burden on patients and caregivers [35], the sustained and stable predictive performance of this model over 4 years is particularly valuable. By delivering reliable risk estimates at multiple time points, the model facilitates shared decision‐making and realistic expectations: low‐risk patients can be spared unnecessary aggressive therapies, frequent visits and the psychological distress of constant uncertainty [36]; high‐risk patients benefit from proactive monitoring and timely interventions for optimized outcomes [37]. Furthermore, the annual event rates and risk differences provide intuitive absolute risk estimates‐for example, an extra 42 or 35 LOA events per 100 person‐years in the high‐risk group, numbers that are immediately understandable to clinicians and parents for clinical communication, resource allocation and trial enrichment. To facilitate clinical translation, the final RSF model is publicly accessible as an interactive web application using Streamlit. Users can input six parameters and instantly obtain an individualized survival curve and predicted LOA risks. This tool may lower the barrier to applying machine learning in routine DMD care, enable rapid what‐if scenarios (e.g., ‘what if NSAA improves by three points?’) and empower physicians and families to make more informed, evidence‐based decisions.

Several limitations should be acknowledged. First, thresholds inferred from SHAP dependence plots should be interpreted as exploratory reference values rather than definitive fixed clinical cutoffs. Feature collinearity can partition SHAP importance across correlated predictors, which may shift observed inflection points when different feature combinations are adopted for model training. All thresholds reported herein were generated exclusively from the final six‐predictor RSF model. These candidate thresholds require independent external‐cohort validation before real‐world clinical implementation. Second, the single‐centre design and moderate sample size may limit generalisability; therefore, external validation in larger, multicentre, multiethnic cohorts is necessary. The moderate sample size may also contribute to the zero events in the low‐risk group and larger cohorts are needed to confirm the model's negative predictive value. Third, the absence of LOA events in low‐risk subgroups during a median 43.5‐month follow‐up likely reflects limited observation time rather than true zero risk; longer follow‐up and external validation are needed to assess long‐term risk. Fourth, time‐varying covariates were not incorporated, which might further improve predictive accuracy. Fifth, although the RSF model was benchmarked against both clinical and ridge‐penalised Cox models, comparisons with other machine‐learning survival approaches (e.g., gradient boosting or deep survival networks) were not performed. Finally, manual segmentation of muscle FF requires trained radiologists; future work should explore automated segmentation to improve reproducibility and facilitate clinical implementation.

In conclusion, we successfully developed and validated a machine learning‐based risk stratification tool that integrates clinical variables and quantitative MRI fat fraction parameters to predict time to LOA in DMD. Our model demonstrated superior predictive performance compared with a conventional clinical model and was deployed as a freely accessible web application (DMD‐TIMER) to facilitate personalized risk assessment. Future efforts should focus on external validation in larger, multicentre cohorts and the development of automated MRI segmentation to enhance clinical applicability.

## Funding

This work was supported by the Clinical Research Funding of Chinese Society of Cardiovascular Disease (CSC) of 2019 (No. HF‐CSC2019B01), Project of Chengdu Science and Technology (2022‐GH03‐00011‐HZ), National Natural Science Foundation of China (Nos. 82572196, 82471970, 824B2052, 82402250, 825B2052), Key Project of Natural Science Foundation of Sichuan Province (2026NSFSCZY0011), Natural Science Foundation of Sichuan Province (2026NSFSC0584) and Chengdu Science and Technology Program (2026‐YF08‐00166‐GX, 2026‐XT00‐00013‐GX).

## Conflicts of Interest

The authors declare no conflicts of interest.

## Supporting information


**Figure S1:** Axial magnetic resonance imaging of the hip and pelvic muscles in a 9‐year‐old boy with Duchenne muscular dystrophy. (A) Four major cross‐sectional levels. (B) Sequences of Dixon (F). (C) Sequences of Dixon (W). (D) T1 maps sequences. (E)T2 maps sequences. i iliacus, ii gluteus minimus, iii gluteus medius, iv gluteus maximus, v tensor fascia lata, vi sartorius, vii pectineus, viii obturator internus, ix adductor longus, x adductor brevis, xi adductor magnus, xii vastus lateralis, xiii rectus femoris, xiv vastus medialis, xv vastus Intermedius, xvi biceps femoris, xvii semitendinosus, xviii semimembranosus, xix gracilis.
**Figure S2:** Screening of quantitative MRI variables by Lasso regression. (A) Lasso Coefficient Path. Each coloured line shows the standardized coefficient of one MRI predictor against the L1 norm. Increasing L1 norm reduces the LASSO penalty λ, allowing variables to enter the model sequentially from left to right (nonzero coefficients). The three final fat‐fraction predictors (rectus femoris FF, vastus lateralis FF, obturator internus FF) are shown in panel C. (B) 5‐fold cross‐validation curve for Lasso regression; the red dashed line denotes λ.min = 0.0586, which yielded 11 MRI features with nonzero coefficients and the blue dashed line denotes λ.1se. λ.min was used for feature selection. (C) Bar chart showing the ranking of selected features by LASSO with λ.min.
**Figure S3:** Screening of variables based on Boruta algorithm. Variables having box plot in green are important, in yellow as tentative and in red as rejected.
**Figure S4:** Screening of variables based on recursive feature elimination with random survival forest results. (A) C‐index curve as a function of feature count, with the optimal feature count (*n* = 3). (B) Top 10 feature importance rankings based on the full random forest survival model.
**Figure S5:** Venn diagram showing the overlap of features selected by LASSO regression, Boruta algorithm and recursive feature elimination (RFE).
**Figure S6:** Time‐dependent AUCs for the RSF, clinical Cox and ridge Cox models in the training and test sets. (A) Training set: the RSF (red), clinical Cox (blue) and ridge Cox (green) models exhibit high discrimination across all evaluated time horizons. (B) Test set: model performance is assessed at 12, 24, 36 and 48 months to evaluate generalizability. Shaded bands represent 95% confidence intervals.
**Figure S7:** Time‐dependent area under the curve for the RSF, clinical Cox and ridge‐penalised Cox models in the test set.
**Figure S8:** Decision curve analysis for the RSF, clinical Cox and ridge‐penalised Cox models in the test set.
**Figure S9:** SHAP and SurvSHAP(t) analysis for model interpretation. (A) SHAP feature importance summary and beeswarm plot. (B) SurvSHAP(t) analysis showing time‐varying feature importance over the follow‐up period.
**Figure S10:** SHAP waterfall plots for representative patients from each risk group. (A) Low‐risk group; (B, C) two representative patients from the intermediate‐risk group; (D) High‐risk group.
**Table S1:** Comparison of baseline characteristics of patients with DMD in the train cohort and test cohort.
**Table S2:** Univariate and multivariate Cox regression analyses for LOA in DMD patients.

## Data Availability

All data supporting the findings of this study are included in the manuscript and are available from the corresponding author upon reasonable request.
