# Supplementary material for: Machine Learning Integrating Quantitative MRI and Clinical Features Predicts Loss of Ambulation in Duchenne Muscular Dystrophy
Source: J Cachexia Sarcopenia Muscle. 2026 Oct 22;17(5):e70413. doi: 10.1002/jcsm.70413 (PMC13690469; doi:10.1002/jcsm.70413)
Supplement: Supplementary file 1 [file JCSM-17-e70413-s001.docx]

**Supplementary figures**

**Figure S1.** Axial magnetic resonance imaging of the hip and pelvic muscles in a 9-year-old boy with Duchenne muscular dystrophy. (A) Four major cross-sectional levels. (B) Sequences of Dixon (F). (C) Sequences of Dixon (W). (D) T1 maps sequences. (E)T2 maps sequences. i iliacus, ii gluteus minimus, iii gluteus medius, iv gluteus maximus, v tensor fascia lata, vi sartorius, vii pectineus, viii obturator internus, ix adductor longus, x adductor brevis, xi adductor magnus, xii vastus lateralis, xiii rectus femoris, xiv vastus medialis, xv vastus Intermedius, xvi biceps femoris, xvii semitendinosus, xviii semimembranosus, xix gracilis.

**Figure S2.** Screening of quantitative MRI variables by Lasso regression. (A) Lasso Coefficient Path. Each coloured line shows the standardized coefficient of one MRI predictor against the L1 norm. Increasing L1 norm reduces the LASSO penalty λ, allowing variables to enter the model sequentially from left to right (non‑zero coefficients). The three final fat‑fraction predictors (rectus femoris FF, vastus lateralis FF, obturator internus FF) are shown in panel C. (B) 5‑fold cross-validation curve for Lasso regression; the red dashed line denotes λ.min = 0.0586, which yielded 11 MRI features with non-zero coefficients, and the blue dashed line denotes λ.1se. λ.min was used for feature selection. (C) Bar chart showing the ranking of selected features by LASSO with λ.min.

**Figure S3.** Screening of variables based on Boruta algorithm. Variables having box plot in green are important, in yellow as tentative, and in red as rejected.

**Figure S4.** Screening of variables based on Recursive Feature Elimination with Random Survival Forest results. (A) C-index curve as a function of feature count, with the optimal feature count (n = 3). (B) Top 10 feature importance rankings based on the full random forest survival model.

**Figure S5**. Venn diagram showing the overlap of features selected by LASSO regression, Boruta algorithm, and Recursive Feature Elimination (RFE).

**Figure S6**. Time‑dependent AUCs for the RSF, clinical Cox, and ridge Cox models in the training and test sets. (A) Training set: the RSF (red), clinical Cox (blue), and ridge Cox (green) models exhibit high discrimination across all evaluated time horizons. (B) Test set: model performance is assessed at 12, 24, 36, and 48 months to evaluate generalizability. Shaded bands represent 95% confidence intervals.

**Figure S7**. Time‑dependent area under the curve for the RSF, clinical Cox, and ridge-penalised Cox models in the test set.

**Figure S8.** Decision curve analysis for the RSF, clinical Cox, and ridge-penalised Cox models in the test set.

**Figure S9**. SHAP and SurvSHAP(t) analysis for model interpretation. (A) SHAP feature importance summary and beeswarm plot. (B) SurvSHAP(t) analysis showing time-varying feature importance over the follow-up period.

**Figure S10**. SHAP waterfall plots for representative patients from each risk group. (A) Low-risk group; (B, C) two representative patients from the intermediate-risk group; (D) High-risk group.

**Supplementary tables**

**Table S1.Comparison of baseline characteristics of patients with DMD in the train cohort and test cohort.**

| Characteristics | ALL (n=188) | Train (n=148) | Test (n=40) | P value |
| --- | --- | --- | --- | --- |
| Age (year) | 9.10 (7.90;10.20) | 9.10 (7.88;10.30) | 9.05 (7.97;9.83) | 0.919 |
| Height (cm) | 1.25 (1.17;1.33) | 1.25 (1.17;1.33) | 1.25 (1.19;1.31) | 0.806 |
| Weight (kg) | 27.00 (22.50;33.00) | 27.25 (22.98;32.62) | 26.25 (22.00;33.00) | 0.351 |
| BMI ( kg/m²) | 17.50 (15.78;19.92) | 17.60 (15.90;20.00) | 17.10 (15.25;19.20) | 0.282 |
| Gene mutations |  |  |  | 0.946 |
| Point mutations | 29 (15.43%) | 23 (15.54%) | 6 (15.00%) |  |
| Duplication | 12 (6.38%) | 9 (6.08%) | 3 (7.50%) |  |
| Deletion | 147 (78.19%) | 116 (78.38%) | 31 (77.50%) |  |
| Gc at baseline |  |  |  | 0.991 |
| No | 68 (36.17%) | 53 (35.81%) | 15 (37.50%) |  |
| Yes | 120 (63.83%) | 95 (64.19%) | 25 (62.50%) |  |
| Fracture |  |  |  | 0.094 |
| No | 173 (92.02%) | 139 (93.92%) | 34 (85.00%) |  |
| Yes | 15 (7.98%) | 9 (6.08%) | 6 (15.00%) |  |
| BMD value | 135.31 (118.72;150.57) | 136.28 (119.05;151.76) | 131.18 (116.30;146.48) | 0.737 |
| T value | -1.26 (1.04) | -1.25 (1.06) | -1.33 (0.97) | 0.618 |
| Z value | -1.40 (1.05) | -1.38 (1.07) | -1.46 (0.98) | 0.654 |
| BMD group |  |  |  | 0.663 |
| Z> -2 | 139 (73.94%) | 111 (75.00%) | 28 (70.00%) |  |
| Z≤ -2 | 49 (26.06%) | 37 (25.00%) | 12 (30.00%) |  |
| MRC% | 72.86 (63.64;80.00) | 73.64 (63.64;80.91) | 71.82 (64.09;79.32) | 0.330 |
| CK | 10.77 (6.67;15.02) | 11.07 (6.89;15.31) | 9.85 (6.63;13.29) | 0.217 |
| CK-MB | 132.00 (83.10;187.50) | 135.59 (83.89;216.37) | 113.00 (71.72;176.84) | 0.420 |
| NSAA Score | 19.00 (9.75;29.00) | 19.00 (10.00;30.00) | 17.50 (6.75;24.50) | 0.114 |
| Tensor Fasc Latae T1 | 1203.54 (984.74;1336.55) | 1243.70 (986.49;1351.78) | 1139.66 (981.07;1229.09) | 0.030 |
| Gluteus Med T1 | 1108.25 (520.01;1355.56) | 1190.17 (563.72;1377.02) | 738.76 (366.51;1224.96) | 0.012 |
| Gluteus Min T1 | 1238.37 (1128.58;1390.76) | 1246.74 (1135.59;1410.72) | 1142.69 (1094.35;1340.39) | 0.033 |
| Adductor Magnus T1 | 410.28 (281.00;1257.51) | 396.63 (286.84;1286.78) | 504.15 (276.90;1190.39) | 0.663 |
| Adductor Longus T1 | 1291.55 (1171.22;1442.51) | 1291.92 (1173.95;1431.00) | 1268.82 (1166.71;1462.72) | 0.930 |
| Gracilis T1 | 1299.85 (1187.04;1466.24) | 1327.61 (1194.89;1470.12) | 1231.14 (1170.11;1368.10) | 0.071 |
| Gluteus Max T1 | 577.47 (293.49;1226.42) | 553.74 (293.49;1253.41) | 668.95 (293.24;1137.87) | 0.600 |
| Semitendinosus T1 | 1279.07 (1188.73;1496.26) | 1291.52 (1194.02;1517.92) | 1265.53 (1175.09;1395.14) | 0.233 |
| Iliacus T1 | 1250.27 (1158.50;1351.99) | 1273.62 (1168.73;1366.42) | 1181.96 (1120.40;1285.70) | 0.006 |
| Rectus Femoris T1 | 1201.60 (737.28;1334.79) | 1207.99 (737.28;1337.25) | 1194.18 (706.22;1298.89) | 0.778 |
| Sartorius T1 | 1325.43 (1229.68;1467.62) | 1343.03 (1229.68;1473.92) | 1303.22 (1234.11;1408.10) | 0.485 |
| Obturator Internus T1 | 1365.14 (1225.41;1544.49) | 1388.08 (1253.58;1549.47) | 1246.93 (1122.76;1395.39) | 0.004 |
| Vastus Lateralis T1 | 1094.91 (358.35;1362.91) | 1115.39 (340.56;1387.91) | 1006.90 (405.11;1335.00) | 0.922 |
| Vastus Intermedius T1 | 1199.65 (556.74;1419.07) | 1190.18 (556.74;1419.07) | 1207.24 (614.25;1406.43) | 0.728 |
| Tensor Fasc Latae T2 | 28.41 (23.12;31.54) | 27.95 (22.82;32.22) | 29.06 (26.12;30.09) | 0.405 |
| Gluteus Med T2 | 30.46 (24.10;53.16) | 30.22 (23.73;47.79) | 34.10 (29.26;61.25) | 0.057 |
| Gluteus Min T2 | 26.52 (21.79;30.25) | 26.52 (21.78;30.02) | 26.00 (23.14;33.34) | 0.324 |
| Adductor Magnus T2 | 42.26 (26.91;93.51) | 41.53 (26.36;89.03) | 50.31 (28.39;110.06) | 0.463 |
| Adductor Longus T2 | 29.48 (24.15;33.45) | 29.22 (23.99;33.03) | 30.34 (25.80;35.34) | 0.093 |
| Gracilis T2 | 25.90 (22.54;29.17) | 25.52 (22.42;28.92) | 27.26 (23.79;29.80) | 0.059 |
| Gluteus Max T2 | 35.56 (27.50;84.23) | 37.95 (26.81;85.14) | 32.45 (28.84;73.08) | 0.945 |
| Semitendinosus T2 | 27.24 (22.40;30.11) | 26.94 (21.97;29.86) | 28.20 (24.14;30.38) | 0.275 |
| Iliacus T2 | 26.74 (22.70;29.85) | 26.56 (22.42;29.54) | 27.88 (23.87;31.20) | 0.042 |
| Rectus Femoris T2 | 29.43 (24.05;45.25) | 29.48 (23.96;45.07) | 29.30 (25.47;58.13) | 0.569 |
| Sartorius T2 | 27.34 (24.01;31.23) | 26.88 (23.75;30.54) | 28.80 (25.74;33.79) | 0.025 |
| Obturator Internus T2 | 28.50 (23.48;32.30) | 27.91 (23.45;32.75) | 29.48 (23.91;31.01) | 0.821 |
| Vastus Lateralis T2 | 31.92 (24.68;60.10) | 30.79 (24.03;60.58) | 35.86 (28.40;58.28) | 0.301 |
| Vastus Intermedius T2 | 29.75 (23.65;38.27) | 29.38 (23.53;38.03) | 29.91 (24.23;45.05) | 0.552 |
| Gluteus Med FF | 31.61 (13.03;61.40) | 27.63 (11.23;62.15) | 42.05 (24.52;60.63) | 0.071 |
| Gluteus Min FF | 8.78 (3.48;31.08) | 7.50 (3.18;29.40) | 13.39 (5.31;38.52) | 0.103 |
| Tensor Fasc Latae FF | 24.05 (7.40;54.01) | 22.34 (8.34;53.65) | 27.56 (5.01;61.21) | 0.709 |
| Adductor Magnus FF | 64.35 (26.01;81.90) | 63.12 (24.89;82.15) | 67.60 (43.82;76.43) | 0.692 |
| Adductor Longus FF | 6.72 (2.24;28.77) | 5.96 (2.24;23.63) | 7.91 (2.70;43.86) | 0.207 |
| Adductor Brevis FF | 12.19 (3.39;51.60) | 11.22 (2.97;53.02) | 21.06 (4.98;50.32) | 0.157 |
| Pectineus FF | 14.15 (3.89;45.51) | 13.77 (3.60;41.89) | 16.72 (6.45;53.31) | 0.307 |
| Gracilis FF | 4.46 (1.78;9.67) | 4.60 (1.78;9.67) | 3.98 (1.96;9.55) | 0.992 |
| Gluteus Max FF | 58.67 (39.60;73.61) | 59.45 (39.37;75.23) | 58.24 (43.95;70.49) | 0.943 |
| Semitendinosus FF | 11.37 (4.31;25.48) | 11.74 (4.27;25.97) | 10.08 (4.87;21.72) | 0.995 |
| Semimembranosus FF | 14.25 (5.00;32.82) | 13.25 (4.90;34.08) | 14.74 (6.70;27.53) | 0.465 |
| Biceps femoris FF | 27.80 (6.68;53.99) | 26.03 (5.64;60.28) | 32.72 (18.20;49.60) | 0.316 |
| Iliacus FF | 4.44 (1.66;11.37) | 4.44 (1.64;11.37) | 4.58 (1.67;11.56) | 0.982 |
| Rectus Femoris FF | 21.52 (6.32;57.27) | 19.78 (5.60;56.14) | 31.63 (9.93;59.90) | 0.286 |
| Sartorius FF | 6.42 (2.82;16.33) | 5.69 (2.81;15.80) | 9.76 (3.25;18.93) | 0.477 |
| Obturator Internus FF | 6.66 (2.80;20.03) | 5.52 (2.36;17.37) | 11.24 (4.81;32.19) | 0.036 |
| Vastus Lateralis FF | 38.12 (10.28;67.35) | 36.68 (9.19;66.09) | 42.27 (15.66;68.64) | 0.274 |
| Vastus Intermedius FF | 24.26 (4.29;58.89) | 18.43 (3.68;58.90) | 41.48 (12.11;56.46) | 0.180 |
| Follow-up |  |  |  |  |
| LOA time (months) | 43.50 (23.00;54.00) | 43.00 (24.50;54.00) | 46.00 (16.00;55.25) | 0.991 |
| LOA |  |  |  | 0.864 |
| No | 122 (64.89%) | 97 (65.54%) | 25 (62.50%) |  |
| Yes | 66 (35.11%) | 51 (34.46%) | 15 (37.50%) |  |
| Abbreviations:DMD, Duchenne muscular dystrophy; BMI, Body Mass Index; GCs, Glucocorticoids; BMD, Bone mineral density; MRC%, Medical research council; CK, Creatine Kinase; CK-MB, Creatine Kinase Myocardial Band; FF, fat fraction; NSAA, the North Star Ambulatory Assessment; LOA, Loss of ambulation. | | | | |

**Tables S2. Univariate and multivariate Cox regression analyses for LOA in DMD patients.**

| Characteristics | Univariate HR (95% CI) | P value | Multivariate HR (95% CI) | P value |
| --- | --- | --- | --- | --- |
| Age | 1.142(1.027-1.269) | 0.014 | 1.006(0.872-1.16) | 0.937 |
| NSAA Score | 0.835(0.801-0.872) | <0.001 | 0.845(0.802-0.891) | <0.001 |
| MRC% | 0.934(0.917-0.952) | <0.001 | 0.990(0.962-1.018) | 0.468 |
| High | 3.323(0.610-18.108) | 0.165 |  |  |
| Weight | 1.016(0.991-1.042) | 0.212 |  |  |
| BMI | 1.042(0.954-1.138) | 0.356 |  |  |
| BMD value | 0.995(0.986-1.003) | 0.234 |  |  |
| T value | 0.768(0.584-1.011) | 0.060 |  |  |
| Z value | 0.798(0.614-1.038) | 0.093 |  |  |
| Z value ≤ -2 | 1.473(0.795-2.728) | 0.218 |  |  |
| Fracture | 1.638(0.589-4.551) | 0.344 |  |  |
| Glucocorticoid use | 1.615(0.86-3.033) | 0.136 |  |  |
| Gene duplication | 0.866(0.275-2.725) | 0.806 |  |  |
| Gene deletion | 0.579(0.295-1.139) | 0.114 |  |  |
| CK | 0.967(0.924-1.013) | 0.157 |  |  |
| CK MB | 1.00(0.999-1.002) | 0.878 |  |  |
| Abbreviations: DMD, Duchenne muscular dystrophy; LOA, Loss of ambulation; HR, hazard ratio; CI, confidence interval; BMI, Body Mass Index; BMD, Bone mineral density; MRC%, Medical research council; CK, Creatine Kinase; CK-MB, Creatine Kinase Myocardial Band. | | | | |
